# Preparation of parenteral nanocrystal suspensions of etoposide from the excipient free dry state of the drug to enhance in vivo antitumoral properties

**DOI:** 10.1038/s41598-020-74809-z

**Published:** 2020-10-22

**Authors:** Brice Martin, Johanne Seguin, Maxime Annereau, Thomas Fleury, René Lai-Kuen, Giovanni Neri, Anita Lam, Marcel Bally, Nathalie Mignet, Yohann Corvis

**Affiliations:** 1 Université de Paris, CNRS, Inserm, UTCBS, Chemical and Biological Technologies for Health Group (utcbs.cnrs.fr), Faculté de Pharmacie, 4 Avenue de l’Observatoire, 75006 Paris, France; 2 Gustave Roussy, 114 rue Edouard Vaillant, 94800 PharmacyVillejuif, France; 3 Université de Paris, CNRS, Inserm, Cellular and Molecular Imaging Technology Platform, Faculté de Pharmacie, 4 Avenue de l’Observatoire, 75006 Paris, France; 4 Department of Chemistry, University of British Columbia, 2036 Main Mall, Vancouver, BC V6T 1Z1 Canada; 5 Department of Experimental Therapeutics, British Columbia Cancer Research Centre, Vancouver, BC V5Z 1L3 Canada; 6 Present Address: Department of Neurological Surgery, Weill Medical College of Cornell University, New York, NY USA

**Keywords:** Drug delivery, Nanotechnology in cancer, Nanoparticles, Pharmacokinetics

## Abstract

Nanoparticle technology in cancer chemotherapy is a promising approach to enhance active ingredient pharmacology and pharmacodynamics. Indeed, drug nanoparticles display various assets such as extended blood lifespan, high drug loading and reduced cytotoxicity leading to better drug compliance. In this context, organic nanocrystal suspensions for pharmaceutical use have been developed in the past ten years. Nanocrystals offer new possibilities by combining the nanoformulation features with the properties of solid dispersed therapeutic ingredients including (i) high loading of the active ingredient, (ii) its bioavailability improvement, and (iii) reduced drug systemic cytotoxicity. However, surprisingly, no antitumoral drug has been marketed as a nanocrystal suspension until now. Etoposide, which is largely used as an anti-cancerous agent against testicular, ovarian, small cell lung, colon and breast cancer in its liquid dosage form, has been selected to develop injectable nanocrystal suspensions designed to be transferred to the clinic. The aim of the present work is to provide optimized formulations for nanostructured etoposide solutions and validate by means of in vitro and in vivo evaluations the efficiency of this multiphase system. Indeed, the etoposide formulated as a nanosuspension by a bottom-up approach showed higher blood life span, reduced tumor growth and higher tolerance in a murine carcinoma cancer model. The results obtained are promising for future clinical evaluation of these etoposide nanosuspensions.

## Introduction

Nanomedicines have paved the way to new opportunities for therapeutic treatments since biocompatible nano-size based particles may enhance the overall pharmacological properties of a given drug substance^1^, such as improved bioavailability and targeting^2^, extended release^3^, decreased side effects^4^, as well as expanding various administration routes^5^. Since the last decade, parenteral nanocrystal (NC) suspensions of active pharmaceutical ingredients (APIs) have emerged for various therapies as it was published by our group as a review 1 year ago^6^. As far as chemotherapy treatments are concerned, many researches lead to the proof of concept for NC preparation and stabilization (e.g. doxorubicin^7^, hesperetin^8,9^, hydroxycamptothecin^10,11^, oridonin^12^, paclitaxel^13–19^, puerarin^20^, riccardin^21^, silybin^20^). However, until the present work, no antitumoral drug has been marketed as a NC preparation for i.v. administration for cancer treatments^6,22–24^. In this study, etoposide (ETO) was prepared for the first time as nanocrystals, using the antisolvent precipitation process. This well-known chemotherapy agent is used against deadliest cancers such as lung, breast, colon or testicular cancer^25^. ETO inhibits the enzyme topoisomerase II which is crucial in controlling the DNA conformational arrangement by generating double-stranded breaks in the DNA molecule^26^. Cell death is induced when sufficient covalent topoisomerase-split DNA complexes are formed and stabilized, giving durable DNA strand breaks, switching cells metabolic activity to apoptosis^27^. In this investigation, agglomerates of ETO NCs were obtained for the first time in a dried powder after slow and complete evaporation of the solvent/antisolvent free of stabilizing agent. Then, the nanocrystals powder was dispersed and stabilized with Pluronic F-127 aqueous solution, allowing a reduction in the non-specific interactions with the immune system, enhancing the in vivo performances. Indeed, as soon as NCs are injected, the proteins present in the bloodstream start binding to the NC surface to form a protein corona. These proteins are acknowledged by the mononuclear phagocyte system (MPS) with rapid clearance of the antigen from the blood. In the present study, stabilization of NCs with F-127 provided a PEG coating advantageous to reduce macrophage interactions and limit the activation of the immune system at least for a single injection^28–31^.

Herein, we report the design, characterization, in vitro and in vivo efficiency of a widely used antitumoral drug substance, ETO, formulated as a NC suspension. For many applications, nanocrystallization main features have to be apprehended and controlled to properly design optimized NCs regarding morphology, size, thermal behavior, stability over time, physical state, dissolution rate and so forth^32^. The effects of stabilizing polymer/drug substance ratio, water/solvent ratio, drug substance concentration and nature of polymer are also discussed. Indeed, these parameters are crucial for optimizing NC formulation and pharmacokinetics^33–36^. The results have been analyzed and compared in order to select the most suitable ETO NC formulation for in vitro and in vivo studies. The size and morphology of NCs were further characterized using scanning and transmission electron microscopies (SEM, TEM). Differential scanning calorimetry (DSC), Powder X-Ray Diffraction (PXRD) and thermal microscopy were used to define the crystallinity of the ETO nanoparticles. In vitro drug release profile evidenced that the ETO NCs provided a sustained release kinetics as compared to the marketed product, i.e. Toposar. In vitro and in vivo cytotoxicity studies of ETO NCs formulation in comparison with Toposar were performed on two cell lines (Carcinoma colon CT26 and 3LL Lewis lung carcinoma cells). The plasma and tissues pharmacokinetics of ETO were performed to determine the fate of the drug after administration in mice. Anticancer efficacy of the ETO NCs formulation and Toposar were tested after i.v. injection in a murine colon carcinoma model. Tumor volumes and mice survival were followed to determine the response to the anticancer treatment. The outcomes in this study evidenced that the delivery of ETO NC suspension, stabilized with merely F-127 0.2% w/v, achieved a better anticancer efficacy in comparison to the marketed product containing high quantities of ethanol and polymer, namely polyethylene glycol 300 (PEG 300; concentration: 3.25% w/v).

## Results

### Design and characterization of the nanocrystalline etoposide

**Figure 1:**
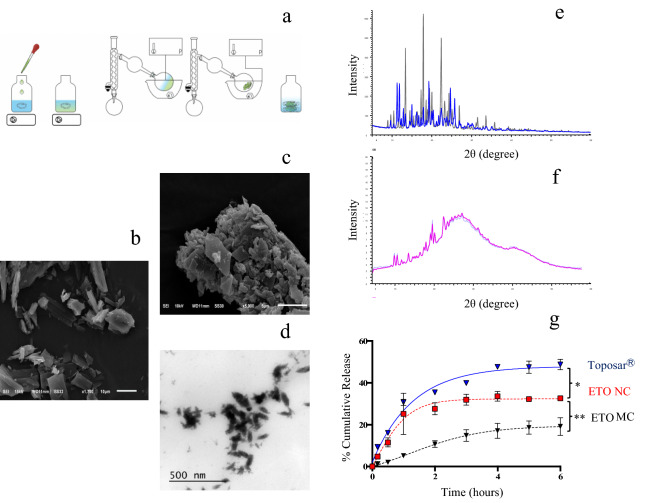
(**a**) Schematic representation of ETO NC preparation using the antisolvent precipitation process. (**b**) SEM image of ETO MC powder, scale bar 10 µm. (**c**) SEM image of ETO NC powder, scale bar 5 µm. (**d**) TEM image of ETO NCs/F-127 0.03% w/v, scale bar 0.5 µm. (**e**) PXRD patterns of ETO MC (blue) and ETO NC powder (black). (**f**) PXRD patterns of ETO NCs in F-127 aqueous solution. (**g**) Release kinetics profiles of Toposar, ETO NCs F-127 0.2% w/v, ETO MC F-127 0.2% w/v in HBS buffer incubating at 37 °C under constant agitation. *p_ETO NC-Toposar_ < 0.0, **p_ETO NC-ETO_ < 0.05.

The antisolvent precipitation technique has been adapted for ETO nucleation optimization (Fig. 1a). The solvent/antisolvent volume ratio, API/excipient mass ratio, and the nature of the surfactant were studied to engineer an ideal NC preparation for in vivo evaluation following systemic injection (Figs. S1, S2 and Table S1 of Supplementary Information). Contrary to conventional NC preparations, the FDA-approved stabilizing agent Pluronic F-127, has been introduced after complete solvent evaporation (Fig. 1a). Physicochemical screening studies of the NC suspensions allowed fixing the optimal ETO/F-127 molar ratio at 11.4 (i.e. 2.5 mg ETO, 5 mg F-127) with the lowest excipient quantity. The characterization by SEM and TEM of the size and morphology of ETO NCs were verified before and after redispersion in water. SEM images show raw ETO powder (Fig. 1b, scale bar 10 µm) compared to ETO powder after the solvent/antisolvent evaporation, i.e. before redispersion (Fig. 1c, scale bar 5 µm). As expected, raw ETO presented particles in the micrometer range (> 10 µm) in comparison with the ETO powder obtained prior to the redispersion which presented agglomerates of nanoparticles of 1 µm or lower confirming the size reduction of ETO after the antisolvent precipitation process. The accurate size of ETO NCs cannot be observed with SEM because of nanoparticle agglomeration. Agglomerates of nanoparticles were also observed with fenofibrate studied by Li et al.^37^. Regarding the morphology of ETO, NC agglomerates exhibited both rod and polyhedral shapes with a predominance for the polyhedral form. As for the raw ETO particles (Fig. 1b), most of them have a rod-like shape with a length up to 30 µm and width up to 10 µm, which tag these particles as microcrystals (MCs). Notably, NCs were originally precipitated and dried without surfactant to obtain a free-stabilizer powder to open up possibilities concerning the NCs self-sheathing. We prioritized the use of a stabilizer during the redispersion only to (i) allow a long-term storage of the raw material as NCs API, and (ii) limit the amount of excipient in the final product formulation. Therefore, the coating of the NCs was obtained during the redispersion process to prevent the NCs from agglomeration for a convenient active delivery nanosystem for the in vivo experiments. Dry ETO NC obtained after redispersion in F-127 0.08% w/v aqueous solution (Fig. 1d) was then imaged by TEM to emphasize the difference between the ETO NC powder and the coated ETO NC. One can argue that in solution, the dispersed NCs are more uniformly reduced to nanosize particles in the range of ~ 100 nm (Fig. 1d); rod and polyhedral morphologies could still be observed. Liu et al*.* had similar shapes for indomethacin and itraconazole NCs obtained by wet milling and stabilized by F-127 or F-68 with a stabilizer concentration superior at 2% w/v^38^.

ETO crystalline structures were obtained by PXRD analysis for the ETO MC powder (Fig. 1e, blue line), ETO NC powder (Fig. 1e, black line) and ETO NC dispersion in solution with F-127 0.03% w/v (Fig. 1f). The objective was to evidence the crystallinity of ETO NC powder after the antisolvent precipitation process and also to verify that NCs are still in the crystal form after redispersion in water. The PXRD pattern obtained for ETO NC powder confirms the total crystallinity of the ETO after processing as sharp peaks can be detected^39^. Even though ETO NC peak profiles can be assigned to the ETO MC powder, minor shifts are observed. This could be possibly explained by a partial formation of another crystalline stacking of ETO. Also, peak intensity is affected by crystallinity, drug crystal size and also merely by the NC powder packing in the well sample holder, the latter is not a reliable parameter to understand the crystallinity of the drug^40^. The diffraction pattern obtained for the ETO was comparable to those reported in the literature^41^. If the ETO nanoparticles are definitely crystalline in the solid state, the redispersion in solution ought to not affect their crystallinity. Indeed, PXRD analysis of ETO NCs in solution was performed and clearly displayed sharp crystalline peaks out of a broad peak corresponding to the diffraction of amorphous matter, i.e. glass capillary and water (Fig. 1f). This result confirmed that ETO NCs kept their crystallinity after redispersion in solution. However, it is surprising that the ETO NCs displayed intense and sharp peaks according to their ~ 100 nm size, hence, the NCs coalescence in the glass capillary could have occurred to unveil such peaks on the diffraction pattern. This hypothesis cannot be verified by the literature as no publications have been interested in the crystallinity of drug NCs after redispersion in solution.

Nevertheless, from the above results, we demonstrate for the first time that it is possible to obtain nanocrystals from etoposide using the bottom-up approach, without stabilizer using the antisolvent precipitation technique. The only attempt with nanosized crystals of ETO available in the literature was published by Merisko-Liversidge et al.^42^. In the latter work, the wet milling top-down approach was performed producing bigger nanoparticles (256.2 ± 53.0 nm), containing a 4 times higher ETO/F-127 molar ratio (45.6) compared to the present study (117 ± 28 nm and 11.4, respectively). Besides, the NCs suspensions prepared by Merisko-Liversidge and co-workers were not stable over time, rendering in vivo applications unsuitable.

### Dissolution study of etoposide nanocrystals

The objective was to compare ETO/F-127 suspensions, namely NCs and MCs as regard to the Toposar considered as the solubilized form of ETO due to its high ethanol and surfactant content. Dissolution study of ETO NCs with F-127 evidenced a reduced dissolution as regard to the marketed product Toposar (Fig. 1g). In contrast, the dissolution measured for ETO NCs was higher than for ETO MCs: 18% of the initial mass was released after 6 h for the MCs (AUC_0–6 h_ (µg.h/mL) = 45.6 ± 2.7), whereas for the NC formulations 35% was released (AUC_0–6 h_ (µg.h/mL) = 103.9 ± 4.0). This is consistent with the smaller size of ETO NCs which exhibit a higher specific area than ETO MC suspension where particles have low specific area (larger particles size > 3 µm) and thus present a lower dissolution rate^43^. Liu et al*.* comparing paclitaxel nanocrystals *versus* the paclitaxel commercialized form Taxol also showed a slower release of the NCs reaching less than 10% in 6 h while 20% of paclitaxel was released from Taxol^15^. Han et al. witnessed comparable dialysis results with hydroxycamptothecin (HCPT) nanosuspension for hepatic cancer therapies. After 1.5 h, conventional product released 100% of HCPT, HCPT MCs released 20% and the HCPT NCs released 40% at the same time, proving that these formulations would also display extended blood circulation lifetime. This is of interest in cancer therapy, as it has been reported that increasing the circulation time of nanoparticles results in an increased accumulation of the nanoparticles in vascularized tumors. Non-structured tumor endothelium underlying the tumor elicits an Enhanced Permeability Retention (EPR) effect^4,10,44,45^. Indeed, when an abnormal tumor develops, an inflammatory response takes place which is defined by a hyper vascularization with poor permeability of surrounding tissues^46,47^. This dilation of the endothelium eases the pathway of nanoparticles to better target cancer tissues by diffusion in some tumors, in particular fastly growing which are more vascularized^48,49^. This reported effect initially allowed Doxil to reach the clinic even though it was later on contested as few studies did not result in significant improvement in human, and the amount of drug accumulated was lower than expected^50,51^. Nevertheless, this contributed to the development of personalized nanomedicine, where patients should be detected for the ability of their tumor to be sufficiently vascularized and permeable to provide the EPR effect as earlier suggested^52^. In the context of this personalized nanomedicine, obtaining anticancer drug under NC form is a key feature to improve drug accumulation within the tumor and optimize the treatment.

### Thermal analysis

**Figure 2:**
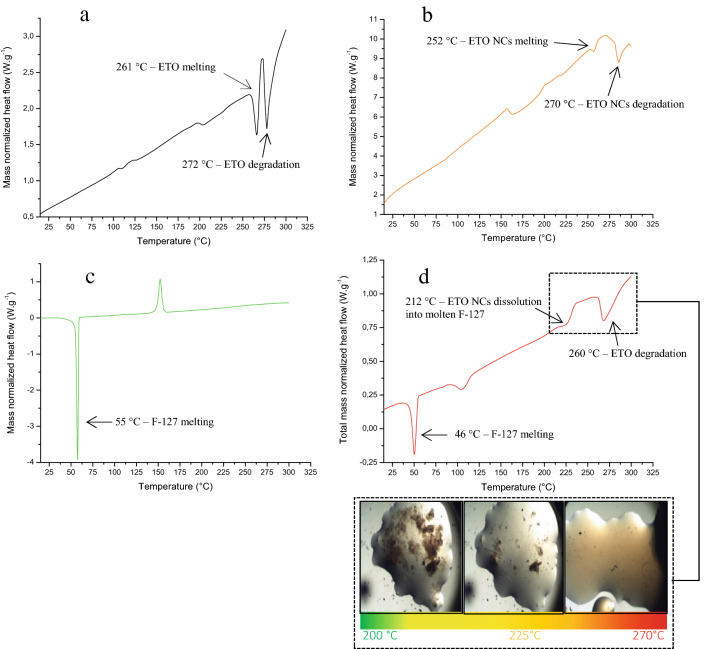
DSC curves for ETO MCs (**a**), free F-127 ETO NCs (**b**), F-127 (**c**), and ETO NCs/F-127 0.03% w/v solid dispersion obtained after evaporation of the continuous medium (**d**). All DSC thermograms are normalized to the total mass sample. Endothermic signals are pointing down. The inset shows the thermal microscopy micrographs as function of the temperature.

Thermal experiments have been performed to compare ETO crystallinity before and after the nanosizing process to confirm the size reduction of ETO NCs and to understand the interactions of the drug with the polymer. The DSC curve of the ETO MC powder (Fig. 2a) exhibited an endothermic peak with an onset temperature of 261 °C corresponding to the ETO melting, and a second endothermic peak at 272 °C due to the ETO degradation. The DSC curve of ETO NC powder without stabilizing agent F-127 exhibited two endothermic peaks with a precocious fusion peak at 252 °C (Fig. 2b). This can be explained by the size reduction of ETO NCs which shifts the melting temperature to a lower temperature. This phenomenon has been demonstrated for the first time in our group for organic compounds such as salicylic acid, benzophenone and acetaminophen^53^. As expected, the degradation signal of ETO NCs takes place nearly at the same temperature as that of ETO MCs (Fig. 2a,b). As far as the thermal behavior of ETO NCs/ F-127 solid dispersion is concerned, the corresponding DSC curve presents a melting peak at about 46 °C corresponding to the melting of F-127 and two other endothermic transformations (at about 97 °C and about 212 °C, cf. Fig. 2d). The depletion of 10 °C of the melting temperature of F-127 in the solid dispersion (Fig. 2d) compared to that of the raw polymer (Fig. 2c) evidences the API/excipient interactions. Indeed, the insertion of NC within the polymeric matrix induces lower polymer chains interactions compared to the raw F-127 compound. This implies a diminution of the temperature/energy needed to melt the polymer mixed with the ETO NCs. At this stage, the signal obtained for the solid dispersion around 97 °C cannot be explained (Fig. 2d). However, the latter signal might be related to the liquidus transition of a possible ETO/F-127 eutectic system. The signal around 212 °C obtained for the ETO NCs/F-127 solid dispersion confirms the nanosized ETO distribution within the polymeric matrix since the temperature of the corresponding signal is lower than that of ETO NCs (Fig. 2d). As for the interpretation of the thermal behavior of the polymer, the melting temperature depletion of the ETO NC proved that the NC/F-127 intermolecular interactions are favored over the NC/NC interactions. These overall differential scanning calorimetry interpretations were confirmed by thermal microscopy experiments, especially for the melting/dissolution process of the ETO NCs into the molten F-127, followed by degradation of the API (inset of Fig. 2d).

### In vitro CT26 and 3LL cytotoxicity

In vitro tests were performed to evaluate the efficiency of ETO NCs and the influence of the amount of surfactant on cytotoxicity. ETO NCs were compared to marketed product Toposar. The viability of CT26 cells was evaluated after 48 and 72 h. The data obtained from the graphical representation of percent viability as function of the ETO NC concentration (Fig. S5 of Supplementary Information) are gathered in Table 1. The inhibitory concentrations for 50% of cells (IC50) after 48 and 72 h for ETO NCs on CT26 cells were 13.73 ± 5.52 and 4.66 ± 0.91 µM, respectively. Essentially, the ETO NCs were as efficient as Toposar (p > 0.05), having an IC50 at 48 and 72 h of 16.71 ± 9.95 and 4.55 ± 0.50 µM respectively. Correspondingly, Lia et al.^15^ showed a cytotoxicity comparable to Taxol when the paclitaxel drug was formulated as NCs. The 3LL cell line (Table 1) showed a higher sensitivity for all formulations tested in comparison with the IC50 obtained for the CT26 cell lines. ETO is known to be very effective against lung cancer cells as their genomic factors are limited (e.g. mutation or down regulation), decreasing drug resistance^54,55^. Indeed, Toshiwo et al*.* evidenced that a reduced expression of topoisomerase II confers a resistance to ETO in lung cancer cell lines issues from a refractory patient^56^. Therefore, an overexpression of topoisomerase II could explain a higher sensitivity to etoposide^57^.

**Table 1:** CT26 and 3LL viability (IC50) after 48 and 72 h for ETO NCs F-127 0.08% w/v, Toposar.

Formulations	CT26—IC50 at 48 h (µM)	CT26—IC50 at 72 h (µM)	3LL—IC 50 at 48 h (µM)	3LL—IC 50 at 72 h (µM)
ETO NCs/F-127 0.08% w/v	13.73 ± 5.52	4.66 ± 0.91	1.17 ± 0.12	1.01 ± 0.24
Toposar	16.71 ± 9.95	4.55 ± 0.50	1.43 ± 0.13	0.75 ± 0.10

For each formulation, the related excipients were separately tested for the cell viability normalization.

### Nanocrystal cellular uptake in CT26 and 3LL cells

**Figure. 3:**
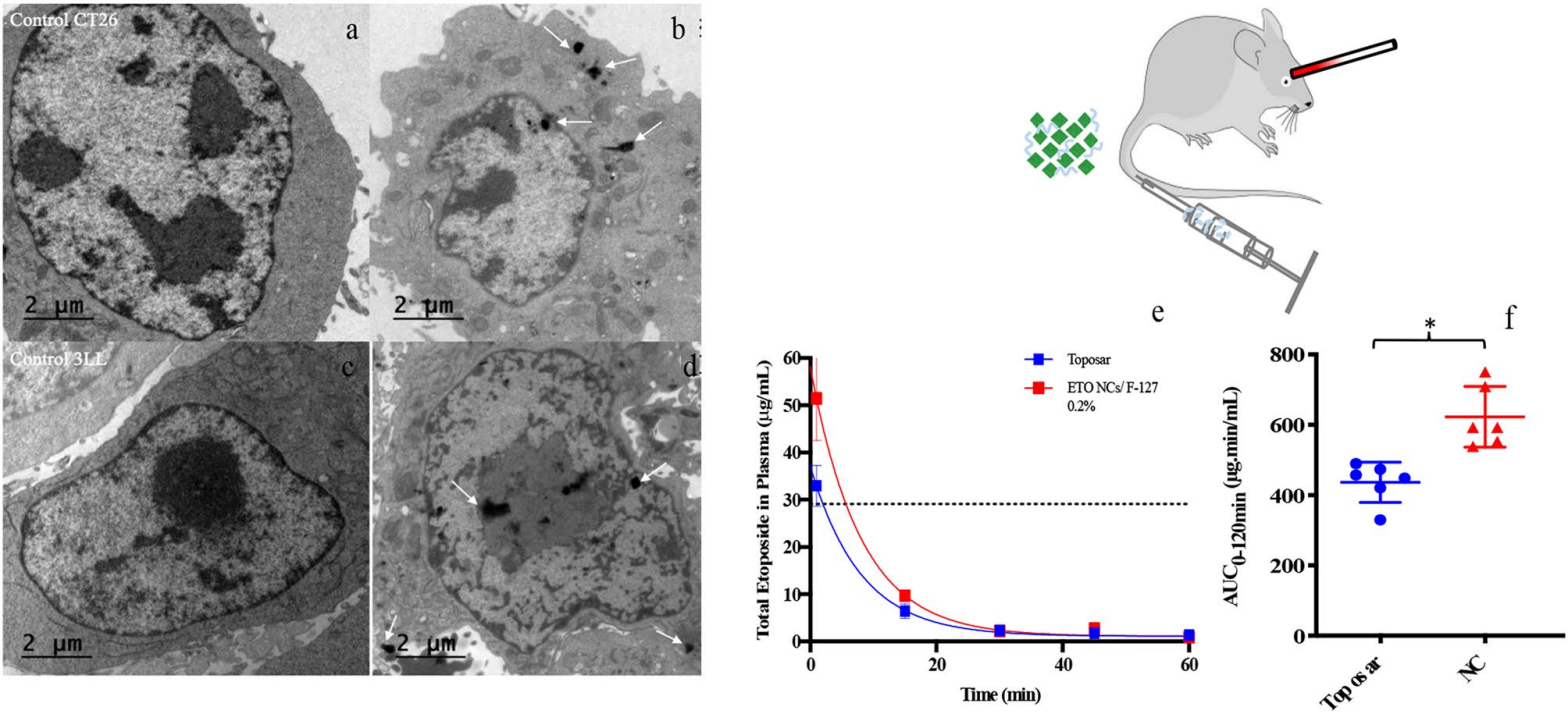
In vitro TEM images of control CT26 (**a**), CT26 after 2 h NCs incubation (**b**), control 3LL (**c**), and 3LL after 2 h NCs incubation (**d**). White arrows show ETO NCs. (**e**) In vivo amount of ETO (µg/mL) in total mice plasma as a function of time after i.v. injection in the mice tail vein. Data shown are means ± SD, n_mice_ = 6, injected dose = 10 mg/kg, i.e. 200 µg per mice. Analysis was fit to a one-phase exponential decay model. (**f**) ETO plasma AUC_0–120 min_ (µg.min/mL). Multiple t-tests with Holm-Sidak method: symbol meaning: *P ≤ 0.05, **P ≤ 0.01, ***P ≤ 0.001. Lack of statistical marking: P > 0.05.

In vitro cell imaging was performed with CT26 (Fig. 3a,b) and 3LL cell lines (Fig. 3c,d) to evidence that cells treated with ETO NCs could undergo apoptosis. Consistently with the toxicity results, we observed cell apoptosis, damaged membrane and smaller size of cells incubated with ETO NCs, indicating that ETO was internalized when formulated as NCs and could maintain its activity. This was expected as nanoparticles are known to be internalized by cells^58–60^. Observation by TEM of black spots with amorphous shape or more definite shape in the hundreds of nanometers could refer to either the internalization of ETO NCs or ETO released from the NCs. Reprecipitation of ETO due to dehydration prior TEM analysis could not be excluded, however ETO precipitates and ETO NCs can be clearly differentiated since needles of etoposide are obtained from precipitation of the solubilized ETO (Fig. S3 of Supplementary Information). Nevertheless, a thorough study should be performed to better define the spots observed after incubation of the NCs with both CT26 and 3LL.

### Plasma pharmacokinetics of etoposide

The ETO plasma concentration profile in mice was assessed for the ETO NCs formulation and Toposar. The pharmacokinetic results are presented in Fig. 3e,f and Table 2. Injection of ETO NC formulations induces significantly higher ETO plasma concentration than the Toposar with an AUC_0–120 min_ almost twofold greater and a higher mean residence time (p < 0.05). This is not the first time that a NC drug form was proved to have a longer lifespan in C57BL/6 mice plasma than its solubilized analog^61^. Ganta et al*.* intravenously injected asulacrine NCs and also perceived a 2.7-fold lifespan enhancement in the plasma compared to the asulacrine solution^62^. Besides the solid form of NCs increasing the plasma lifetime^63^, the use of stabilizer comprising PEG is known to reduce protein binding and therefore extend the particle’s plasma concentration^64,65^. Interestingly, the AUC_0–120 min_ for free etoposide is close to that of Toposar and NCs with the different stabilizer layers present close AUC_0–120 min_ (cf. Supplementary Information, Fig. S4 and Table S2). In addition, the y-axis intercept of the one-phase exponential decay at x = 0, namely Y_0_, is 58.2 µg/mL for ETO/F-127 while 37.2 and 37.6 µg/mL for Toposar and free ETO, respectively (Fig. S4 and Table S2). This proves that the complete nanocrystalline form of etoposide remains in the bloodstream several minutes after the injection, then may start slowly releasing free ETO. To complete such a study, tissues biodistribution analysis was also performed after injection of ETO NCs or Toposar and are detailed in the Supplementary Information (Tables S3 and S4).

**Table 2:** Pharmacokinetic parameters of etoposide for ETO NCs F-127 0.2% w/v, Toposar: maximum amount (µg/mL) in total mice plasma ± SD (n = 6), area under the curve (AUC ± SD (n = 6)), half time (t_1/2_), and mean residence time (MRT ± SD (n = 6)) based on C_theo=0_ = 200 µg. *p_ETO NC-Toposar_ < 0.05.

Formulations	Maximum amount in total mice plasma (µg/mL)	AUC_0–120 min_ (µg min/mL)	Half-time (min)	MRT (min)
ETO NCs/F-127 0.2% w/v	58.2 ± 1.9	608.0 ± 66.8*	5.5	3.04 ± 0.33
Toposar	37.2 ± 1.1	436.0 ± 42.6*	5.5	2.18 ± 0.21

### Anticancer efficacy

**Figure 4:**
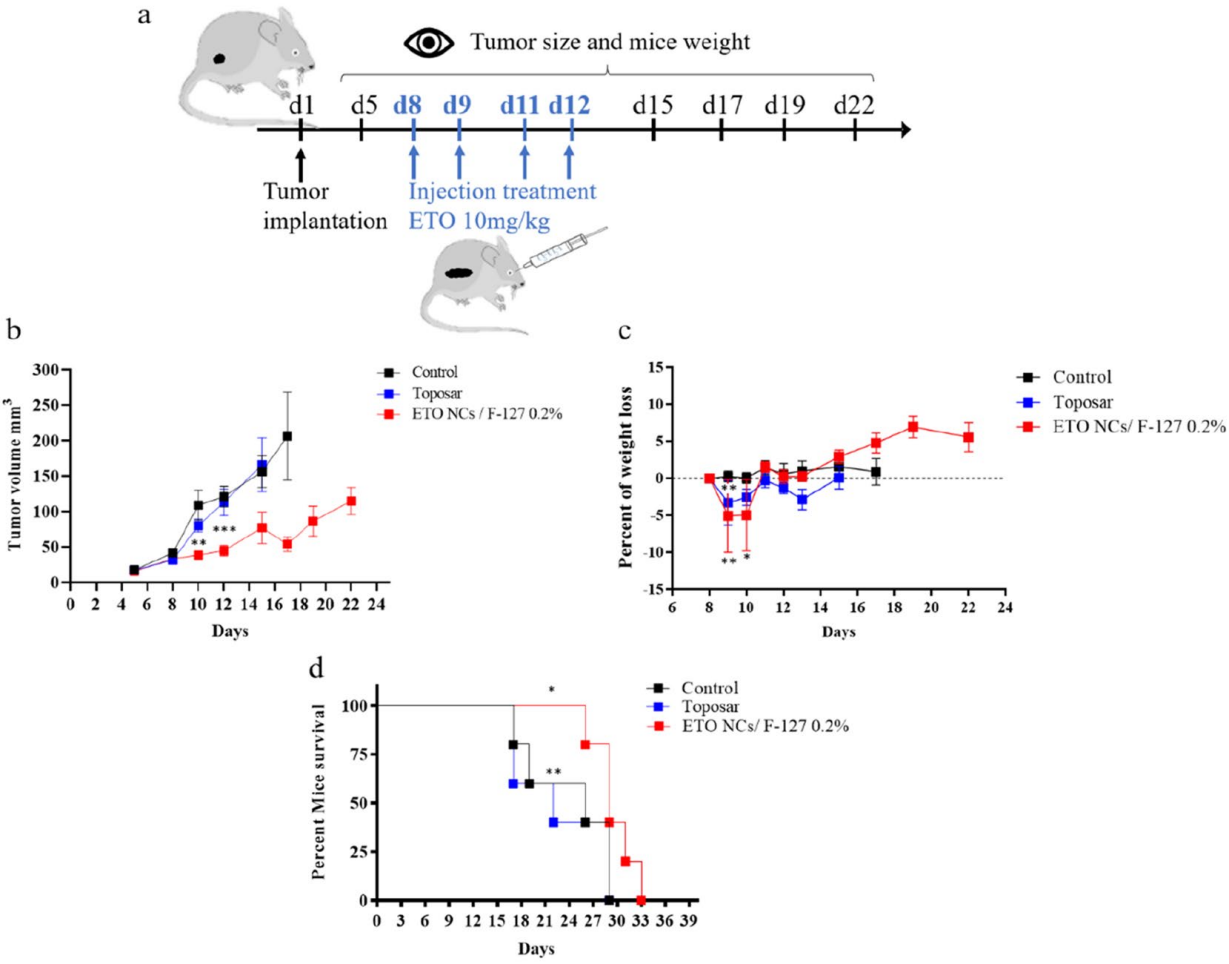
(**a**) Schedule treatment for Balb/c female mice implanted with CT26 subcutaneous tumor. (**b**) Tumor growth inhibition for ETO NCs F-127 0.2% w/v, Toposar and control. Blue arrows show the days of i.v. administration. (**c**) Percent of weight loss of Balb/c mice induced by the ETO anticancer treatment. Weight was estimated every two days and expressed as a percentage of weight at day 8 of the experimentation, *p_ETO NC-Toposar_ < 0.05. (**d**) Kaplan–Meier survival curve for Control, Toposar, ETO NCs F-127 0.2% w/v. Multiple t-tests with Holm-Sidak method: symbol meaning: *P ≤ 0.05,**P ≤ 0.01, ***P ≤ 0.001. Lack of statistical marking: P > 0.05.

The tumor growth inhibition following ETO NCs and Toposar treatments was evaluated on BALB/c female mice (Fig. 4a). Mice received 4 injections of ETO at 10 mg/kg, an injection daily for two consecutive days, a day of rest, followed by an injection daily for two days. The treatment started after 8 days and the tumor growth inhibition was compared for ETO NCs F-127 0.2% w/v and Toposar. The tumor growth inhibition is shown with the complete groups. As soon as one mouse had to be sacrificed due to ethical reasons (in particular, too high tumor volume in the control group), the tumor volume cannot be assessed anymore, and the survival rate is therefore determined. As can be seen in Fig. 4b, for both formulations tested, tumor inhibition was equivalent up to 12 days (p > 0.05). However, after 15 days, ETO NCs/F-127 0.2% w/v were more effective than the marketed product Toposar (p < 0.05). The tumor volume was further decreased after 17 days (3 days after the last ETO injection) in comparison with the Toposar (p < 0.05). Similar results were obtained by Liu et al*.* with a taxane chemotherapy: tumor inhibition effect of paclitaxel NCs was better than Taxol injected intravenously at a concentration of 10 mg/kg^15^. Hypothetically, this could be justified by the size of the NCs which are suitable for blood vessels extravasation and provides a longer bloodstream lifespan. It has also been proven that nanoparticles have a better penetration into the surrounding interstitium of the tumor leading to a better bioavailability and thus anticancer efficacy, in particular for hydrophobic drugs which will be able to enter the cells after release^66,67^. For ETO NCs/F-127 0.2% w/v, the time to reach the median tumor volume in comparison with control was delayed by 5.3 days (Table 3). Toposar slightly postponed the median tumor volume of about half a day revealing a poor treatment response for this formulation (Table 3). Regarding the mice weight loss (Fig. 4c) throughout the investigation, ETO NCs /F-127 0.2% w/v induced a minor weight loss of 0.83% proving that this treatment was well tolerated. For Toposar, a decrease of 2.86% was measured, reflecting the systemic toxicity of the drug. Also, a higher concentration of toxic non-API constituents could foster unwanted side effects causing loss of appetite and inducing mice weight loss^68^. Mice that did not receive treatment and the Toposar-treated cohort were sacrificed at day 17, expressing the virulence of the CT26 colon tumors and the inefficiency of Toposar against that tumor (Fig. 4d). ETO NCs/F-127 0.2% w/v prolonged the median survival time in comparison to Toposar by 6 days (p < 0.05 vs. Toposar by Log-rank test)^69^. Despite a low pharmacokinetic improvement, we found that ETO NC formulation had a better antitumor efficiency than Toposar, probably explained by an enhanced accumulation of the NCs in the CT26 tumor. As regard to the etoposide form after administration, free etoposide is present in the blood stream from the NC formulation. The amount is equivalent at least to ETO solubility at neutral pH and 37 °C. The solubilized ETO in the plasma could explain why there is just a slight improvement of the pharmacokinetic. Nevertheless, it has also been demonstrated in this study that the complete nanocrystalline form of etoposide remains in the bloodstream several minute after the injection, inducing a difference in the therapeutic efficiency of the NC form compared to the Toposar and to the free ETO formulations (Fig. S4 and Table S2). This can be supported by the fact that etoposide amount injected in the mice (0.20 mg per mouse) was higher than the estimated etoposide solubility of 0.14 mg per mouse taking into consideration a mean volume of blood of 1.2 mL in mice. All these data are in accordance with the significant tumor delay noticed which can be attributed to the NC formulations of etoposide. Indeed, Ye and coworkers have demonstrated that when the NCs are totally dissolved, the nanosuspension will mainly show a solution-like behavior for the release, the in vitro cytotoxicity and the in vivo pharmacokinetic experiments^70^. Hence, the results obtained in the present study comfort a mixture of the ETO solubilized and NC forms, in the NC formulation, which is also consistent with the highest amount of ETO recovered in the liver 45 min after injection of the NC formulation (Table S3 of Supplementary Information). As previously mentioned, the NC form was designed in order to increase the accumulation of etoposide within the tumor thanks to the enhanced permeability and retention effect observed with the CT26 which exhibit a higher vascularization^71^. Enhanced antitumor efficacy was also shown in other studies reporting solubilized form versus NCs form of anticancer drugs^15,69,72^.

**Table 3:** Antitumoral effect of ETO NCs F-127 0.2% w/v, Toposar and Control.

Formulations	Body weight variation in % (day of nadir)	Median tumor volume (mm^3^ on day 12)	T/C (%)	Time for median tumor to reach 100 mm^3^ (day)	T-C (day)	MST (day)
Control	–	108	–	9.5	–	26
ETO NCs/F-127 0.2% w/v	− 0.83 (d9) + 4.79 (d17)	47	44	20.6	11.1	29
Toposar	− 2.86 (d13) + 0.41 (d17)	99	92	12.1	2.6	22

ETO dosage: 10 mg/kg. Administration schedule: d8, d9, d11, and d12. Tumor growth inhibition ratio (T/C) = MTV_treated groups_/MTV_control_. Tumor growth inhibition delay in comparison to control (T − C) = TMT_treated groups_ − TMT_control_. MTV, TMT and MST stand for median tumor volume, time for median tumor and median survival time, respectively.

## Conclusion

In this study, a bottom-up approach for ETO nanocrystal-based technology has been developed for the first time to prepare a final product of pharmaceutical interest for parenteral administration and evaluate the therapeutic efficiency. The NC formulations have been optimized to offer better physical and chemical stabilities over time, high drug loading and safety compared to the conventional Toposar and the only reported top-down approach for ETO NC formulation^42^. To engineer ETO NCs, a bottom up technique has been utilized, allowing the control of the crystallization process and achieving nanoparticle formulations with crucial features such as the size, for upcoming in vitro and in vivo studies. Indeed, the size of the NCs impacts their dissolution rate and therefore their in vivo performances. The drug nanoparticle size has to be managed in accordance with the application. Surfactants play an important role regarding etoposide release from the NCs according to their interaction with the nanoparticles surface; they also prevent the NCs coalescence and affect the drug recognition by the immune system^73^. In the frame of this study, Pluronic F-127 was found as the best stabilizing and protecting agent for keeping ETO NC dispersions stable but also injectable in mice for therapeutic purpose. Interestingly, it has been demonstrated that ETO NCs can be directly obtained and stored for several months in the dry form prior to extemporaneous re-dispersion in Pluronic F-127 solution followed by i.v. administration. In this case, long-term stability issues of the solid nanodispersions is consequently bypassed. As far as the therapeutic efficiency of the ETO NCs is concerned, it has been established that the validated formulation is more effective and safer than Toposar. Polymer-coated NC delivery offered better bloodstream lifespan and drug tolerance in a murine carcinoma cancer model. An improved tolerance and efficacy were obtained with ETO NCs (T/C = 44%, i.e. in the range of the minimum level for activity threshold^74^ in comparison with Toposar (T/C = 92%), with the benefit of using 4 times lower mole quantity of stabilizing polymers in comparison with Toposar for the same ETO administrated dose. All these conclusions highlight the benefit of NC strategy of poorly soluble compounds for i.v. dosage forms and provide an easy process to produce and conserve NCs. Furthermore, NC formulations may be adapted to numerous therapeutic matrices such as hydrogels, fibers, and lipidic carriers in order to broaden the nanomedicine development for cancer therapies.

## Methods

### Materials

ETO (> 99% purity, 33419-42-0) was purchased from Santa Cruz Biotechnology (United States of America) and Pluronic F-127 was purchased from Sigma Aldrich (St. Louis, Missouri, United States). MeOH and dimethyl sulfoxide were purchased from Fisher Scientific (Waltham, Massachusetts, United States). Toposar was gifted from Gustave Roussy Hospital (Ivry-sur-Seine, France). Methanol and dimethyl sulfoxide were purchased from Fisher Scientific (Waltham, Massachusetts, United States). All products and solvents were used without further purification. Deionized water (by Milli-Q, filtered through 0.2 μm membrane) was used for the current study. 0.050 μm Whatman Nuclepore polycarbonate membranes used for filtration were purchased from Thermo Fisher Scientific. CT26 and 3LL were purchased from American Type Culture Collection (ATCC, CRL-2638, LGC Standards, Molsheim, France) and cultured at 37 ^∘^C in a 5% CO_2_-humidified atmosphere in Dulbecco’s Modified Eagle Medium (DMEM, Gibco) containing 10% fetal bovine serum (FBS, Gibco Life technologies), 100 μM of streptomycin, and 100 U/mL of penicillin. Isoflurane for anesthesia was purchased from Centravet (France).

### Nanocrystal preparation

The ETO NCs were prepared by the method of antisolvent precipitation (Fig. 1a). Merely 2.5 mg of ETO was dissolved in 1.5 mL absolute methanol (MeOH) in a glass vial and slowly injected under agitation (1200 rpm) in 10 mL of water. The solubilized drug was precipitated by evaporating the entire solution using a rotavapor under vacuum in order to reach a 60 mbar pressure, followed by a decrease to 10 mbar during 30 min. The resulting powder was kept under vacuum to remove any traces of solvent. Then, to engineer the NC suspension, the dry powder was hydrated with a F-127 aqueous solution upon 10 min. sonication. The final NC preparation always contained 2.5 mg ETO plus 5 mg F-127, that is to say an ETO/F-127 molar ratio of 11.4. The in vitro and in vivo experiments were thus performed with NC suspensions prepared with this API/stabilizer ratio, but with different water volumes of redispersion as a function of the application. This led to formulations with 3 different weight volume percentages of F-127 (i.e. 0.03, 0.08 and 0.2% w/v, respectively). The size and shape of the NCs was equivalent for these 3 formulations and were conditioned during the precipitation/evaporation process (*cf.* Supporting information). Noteworthy ETO concentrations for the related control experiments were properly adjusted.

### Etoposide nanocrystals particle size and morphology

The morphological evaluation of NCs has been investigated by a scanning electron microscope (SEM) (Philips XL 30 microscope, Hillsboro, USA). Raw ETO MC powder and processed NC powder were placed on a double-sided tape, then coated with a 30 nm layer of gold under vacuum (10^–6^ Pa) for 2 min, then observed using SEM at an accelerating voltage of 15 kV under vacuum. Also, transmission electron microscopy (TEM) was performed to understand the surface morphology and structure of ETO NCs from the dispersion in the aqueous polymeric solution. A drop of the ETO NCs solution was put on a copper grid with Formvar films Cu 200 Mesh. Then, negative staining was performed by adding a drop of uranyl acetate solution (1% w/v). The excess fluid was removed with filter paper. The grids were examined under a transmission electron microscope (JEM-100S, JEOL, Tokyo, Japan) at accelerating voltage of 80 kV.

### X Ray powder diffraction experiments

The PXRD patterns for ETO NC solutions were obtained using a Bruker APEX DUO diffractometer mounted with a IµS microsource and APEX II CCD Detector with CuK⍺ radiation. The ETO solution was set in a 0.7 mm capillary and flame sealed before mounting. The results were gathered as three still frames to detect 2*θ* from 5 to 60° with 300 s exposures and analyzed using the Bruker XRD2 Eval and Bruker EVA softwares. The PXRD examinations of ETO MC and NC powders were made using a Bruker D8-Advance X-ray diffractometer equipped with a LynxEye silicon strip detector. A copper source was used with a nickel filter leaving CuK⍺ radiation. The generator was set at 40 kV and 40 mA. The samples were scanned from 5 to 60° in 2*θ* with a step size of 0.03° and a counting time of 0.6 s per step. The data was evaluated using Bruker EVA software.

### Dissolution study of etoposide nanocrystals

In vitro release analysis was performed to compare the dissolution rate of ETO NCs/F-127 0.2% w/v with the microcrystals (MCs) from VP-16 powder dispersed in water with 0.03% w/v, and also ETO in Toposar formulation in which ETO is in its solubilized state. In vitro release of ETO NCs was assessed by the dialysis bag diffusion technique. The ETO solutions (4 mL) were placed in a cellulose dialysis bag (molecular weight cutoff of 12.4 kDa) and sealed at both ends using dialysis tubing closure. Then, the dialysis bag was immersed in a compartment containing 36 mL of HEPES buffered saline (HBS) medium (20 and 150 mM HEPES and NaCl, respectively), pH 7.4, which was stirred at 60 rpm and maintained at 37 °C for 6 h. The receptor compartment was covered to prevent the evaporation of the continuum medium. Aliquots (1 mL) were withdrawn at 10 min, 30 min, 1, 2, 4, 6 h, and the same volume of fresh HBS was added to the medium in order to maintain its overall volume at 40 mL after each sampling smear. Then, the samples were analyzed using a Cary 100 Scan UV–visible spectrophotometer (Pittsburgh, USA) set at 283 nm.

### Differential scanning calorimetry and thermal microscopy

The thermal behavior of marketed ETO powder, ETO nanostructured powder without F-127, ETO NCs/F-127 0.03% w/v dried solid dispersion and raw F-127 powder were analyzed by a differential scanning calorimetry (DSC) technique using a DSC3 from Mettler-Toledo (Greifensee, Switzerland). Each sample with a known mass was introduced in an aluminum pan that had been sealed afterward. An empty aluminum pan was used as reference. All experiments were performed in the temperature range from 0 to 300 °C at a 5 K/min scan rate under a 50 mL/min dry air flow in order to homogenize the temperature within the oven. ETO NCs/F-127 0.03% w/v solid dispersions were observed as a function of the temperature by means of an LTS 420 Linkam heating cell (Microvision Instruments, Evry, France) placed under a SMZ 168 microscope (Motic, Kowloon, Hong Kong). Temperature ranged from 23 to 300 °C at 5 K/min. The cooling system was achieved using a T95-HS Linkam device with liquid nitrogen automatically flowing through the cell. The pictures were taken each 5 s (~ 0.42 °C) with a Moticam 2500, 5.0 M pixels, from Motic.

### In vitro CT26 and 3LL

In vitro studies were performed to assess the potential of ETO NCs on cancer cells and to lead future in vivo studies. Cytotoxicity studies of ETO NCs/ F-127 0.08% w/v and Toposar (2 mg citric acid anhydrous, 650 mg polyethylene glycol 300, 80 mg polysorbate 80 and 33% v/v absolute ethanol) were tested on CT26 colon cancer and 3LL Lewis lung cancer cells. Protocol is described for CT26, equivalent for 3LL. First, CT26 colon carcinoma cells were cultured in Dubelcco’s modified Eagle’s medium (DMEM) containing 10% fetal bovine serum and penicillin/streptomycin (50 mmol) at 37 °C. Cells were plated at the concentration of 200,000 cells/mL in 96 well plates for 24 h. Then, CT26 cells were incubated with ETO NC formulation and Toposar. After 48 and 72 h, the tested formulations were removed from wells and cell viability was performed using the colorimetric MTT test^75^. Absorbance was determined at 562 nm in a microplate reader (BioKinetics Reader, EL340). The results are displayed as a percentage of viable cells and the inhibitory concentration for 50% of cells (IC50) was calculated (Fig. S4 of Supporting information).

### Nanocrystal cellular uptake CT26 and 3LL

In vitro TEM imaging studies were performed to observe the internalization of the ETO NCs inside the cells. ETO NCs/ F-127 0.08% w/v was tested on CT26 and 3LL cancer cells. Cells (2.10^5^ cells/mL) were put in a 25 cm^3^ culture flask for 24 h until confluence. Then, CT26 cells were incubated with each ETO NC formulation for only 2 h. After this period, cells were trypsinized (trypsin-ethylenediaminetetraacetic acid (EDTA) 0.5%) and recovered in Falcon tubes. Cells were water washed and fixed with paraformaldehyde 2% + glutaraldehyde 2.5% + Na cacodylate 0.1 M (pH 7.3) + CaCl_2_ 5 mM. Samples were post-fixed in 1% OsO_4_ and stained with filtered (0.22 µm) uranyl acetate 1%. Then, specimens were rinsed in 0.1 M phosphate buffer and dehydrated in an escalating streak of ethanol at 30, 50, 70, 95 and 100% (3 × 10 min. for each) and passed on with propylene oxide and ethanol (50/50 v/v mixture) for 10 min. Followed by polymerization in Epon at 60 °C for 72 h. Samples were sliced (80 nm) using a Leica ultracut S ultramicrotome fitted with a diamond knife^76^. The selected cell sheets were not additionally stained to avoid precipitates that could be confound with ETO NCs. Specimens were studied under a transmission electron microscope (JEM-100S, JEOL, Tokyo, Japan) at accelerating voltage of 80 kV.

### Animals

BALB/c female mice (6 weeks) were supplied by Janvier Laboratory (Le Genest Saint Isle, France). All of the animals were acclimatized at a temperature of 23 ± 2 °C and under light/dark conditions for 4 weeks and provided food and water. All the experiments on rodents were performed in accordance with Guidelines for Care and Use of Laboratory Animals of European directive No. 2010-63, and national guidelines, French decree No. 2013-118. The experiments were also validated by Paris Descartes Ethics Committee for Animal Experimentation, CEEA No. 34 at Paris University under the project number APAFiS #20869.

### Plasma and tissues pharmacokinetics of etoposide

Twelve BALB/c female mice (6 weeks) were used for the determination of the ETO concentration over time in the plasma and selected tissues (liver, spleen, kidneys, lungs). Two formulations were tested, ETO NCs/ F-127 0.2% w/v and Toposar (n = 6 for each formulation). Each formulation was given intravenously at 10 mg/kg ETO. Then, retro-orbital blood samples were performed (200 µL) at different times (1, 15, 30, 45, 60 and 120 min) and added in an Eppendorf tube containing 20 µL of EDTA. Plasma was collected by centrifugation at 2000 rpm for 15 min. and frozen at − 20 °C for further high-performance liquid chromatography (HPLC) analysis. Samples were centrifuged and chloroform was totally evaporated in glass vials. Dry residues were redispersed in 130 µL of acetic acid 1% in water/methanol mixture as a mobile phase (58/42 v/v) and ready for analysis. The HPLC was set as reversed phase (RP-HPLC, 1260 Infinity, Agilent) with isocratic conditions. The analytical column was standard with a reversed phase C18 (150 mm × 4.6 mm, 2.7 μm, Macherey Nagel). The injected volume was 50 μL for all samples.

### Antitumoral efficacy

Fifteen BALB/c female mice (Janvier, St Genest de Lisle, France) aged of 6 weeks were divided into 3 groups (3 × n_mice_ = 5); 2 groups received 2 different ETO formulations, ETO NCs/F-127 0.2% w/v or Toposar and 1 group was used as control. The first 2 groups received 4 injections of ETO at 10 mg/kg, an injection daily for 2 consecutive days, a day for rest, followed by an injection daily for 2 days. The untreated control group was used as comparison for tumor volume. Murine carcinoma CT26 tumors were implanted subcutaneously on day 1 using a 12-gauge trocar (38 mm) into the mouse flank previously disinfected with alcohol. After 8 days, mice were separated in groups in order to have homogeneous tumor volumes. The anticancer treatment started on day 8 as described above to have homogeneous tumor growth in each group. Tumor size and body weight were evaluated using a digital caliper every two days until day 17. Tumor volume (V) was calculated as followed^77^: V = (Length × Width^2^)/2. For the survival study, weight loss superior to 10% or tumor size > 10% of the mice body weight were established as endpoints. All mice were anesthetized before ETO injection in an induction chamber under a flow of oxygen/isoflurane (30/70) (Tec 7, Minerve, Carnaxide, USA).

### Statistical analysis

The plasma and tissues pharmacokinetics, as well as anticancer efficacy data are shown as mean ± standard deviation (SD). Statistical investigation was done by two-way analysis of variance (ANOVA) test with Bonferroni correction with GraphPad Prism version 7. More precisely, plasma and tissues pharmacokinetics analysis were fit to a one-phase exponential decay and a Gaussian model respectively*.* Anticancer efficacy analysis was fit to a baseline corrected model. In vitro cytotoxicity studies were fit to a sigmoidal dose response model and submitted to a one-way ANOVA test. Lack of statistical marking (*) for the in vitro and in vivo experiments indicates that no significance was found.

## Supplementary information

Supplementary Information.
