# Supplementary material for: Preparation of parenteral nanocrystal suspensions of etoposide from the excipient free dry state of the drug to enhance in vivo antitumoral properties
Source: Sci Rep. 2020 Oct 22;10:18059. doi: 10.1038/s41598-020-74809-z (PMC7581827; doi:10.1038/s41598-020-74809-z)
Supplement: Supplementary file 1 — Supplementary Information. [file 41598_2020_74809_MOESM1_ESM.docx]

*Supplementary Information*

**Preparation of parenteral nanocrystal suspensions of etoposide from the excipient free dry state of the drug to enhance *in vivo* antitumoral properties**

Brice Martin^1,†^, Johanne Seguin^1^, Maxime Annereau^2^, Thomas Fleury^2^, René Lai-Kuen^3^, Giovanni Neri^1^, Anita Lam^4^, Marcel Bally^5^, Nathalie Mignet^1^ & Yohann Corvis^1^

^1^Université de Paris, CNRS, Inserm, UTCBS, Chemical and Biological Technologies for Health Group (utcbs.cnrs.fr), Faculté de Pharmacie, 4 avenue de l’Observatoire, 75006 Paris, France. ^2^Gustave Roussy, Pharmacy, 114 rue Edouard Vaillant, 94800, Villejuif, France. ^3^Université de Paris, CNRS, Inserm, Cellular and Molecular Imaging Technology Platform, Faculté de Pharmacie, 4 avenue de l’Observatoire, F-75006 Paris, France. ^4^University of British Columbia, Department of Chemistry, 2036 Main Mall, V6T 1Z1 Vancouver, British Columbia, Canada. ^5^British Columbia Cancer Research Centre, Department of Experimental Therapeutics, BC V5Z 1L3 Vancouver, British Columbia, Canada. ^†^Current affiliation: Department of Neurological Surgery, Weill Medical College of Cornell University, New York, NY, USA. Correspondence and requests for materials should be addressed to Y.C. (email: yohann.corvis@u-paris.fr)

**Nanocrystal Preparation**

The antisolvent precipitation is based on the addition of solubilized drug from organic phase into aqueous solution under agitation, therefore nucleation and nanocrystal formation strongly depend on the organic phase nature and the solubility of the active pharmaceutical ingredient (API) in the aqueous phase. Indeed, the appearance of nuclei occurs when the organic phase is mixed into the aqueous phase, then the nucleation is governed by the diffusion of these nuclei to the liquid interface in the solution monitoring the nanocrystal (NC) properties^1^. It is crucial to select an organic phase miscible to the aqueous solution, easily removable from the mixture and where the drug is highly soluble to ensure homogeneous NCs properties. Here, methanol (MeOH) was preferred as ETO is highly soluble in this mixture. Basically, the drug was solubilized in MeOH and then added to an aqueous solvent containing low amounts of surfactants or no surfactant. The entire solution was completely evaporated to recover a powder containing the drug with or without stabilizer. The powder obtained could be stored for months. The redispersion was performed in a solution where drug is poorly soluble to form a nanosuspension. The striking parameters for the preparation of drug NCs using antisolvent precipitation approach are the solvent/antisolvent volume ratio, the nature of these solvents, the concentration of surfactant and the evaporation rate. The physical properties of drug NCs are directly correlated to these factors. Firstly, API/surfactant ratio has been specially studied to find the most suitable ETO NC formulations. Based on the literature, Pluronic F-127 was used as surfactant and water as antisolvent solution. More precisely, ETO was dissolved in absolute MeOH in a glass vial and slowly injected under agitation in water with or without stabilizer. For further clarity, the quantity of F-127 solubilized in water used during the antisolvent precipitation process is denoted P1. The solubilized drug was precipitated by evaporating the entire solution using a rotavapor under vacuum in order to reach a 60 mbar pressure, followed by a decrease to 10 mbar during 30 min. The resulting powder was kept under vacuum to remove any traces of solvent. Then, to engineer the NC suspension, the dry powder was hydrated with a F-127 aqueous solution upon 10 min. sonication. The quantity of F-127 solubilized in water used during the redispersion process is denoted P2.

Influence of the solvent/antisolvent volume ratio

Notably, the volume ratio of MeOH/H_2_O used for the antisolvent precipitation process is also essential for the preparation of convenient nanodispersions. Indirectly this relies on the concentration of the drug in the organic phase when the mixing with the aqueous phase occurs. The drug nucleation to NCs depends on its initial concentration in the organic solvent as the diffusion of the molecule to the liquid interface (solvent/antisolvent mixture) also changes with the amount of drug. As shown in Fig. S1, an increase of ETO NC size is observed with a diminution of the MeOH/H_2_O volume ratio. With a 1.5/2 ratio ETO NC size was around 400 nm while a 1.5/10 ratio provided ETO NCs around 150 nm. Once again, this may be explained by the fact that the more the ETO is diluted in the mixture, the more its dispersion, changing the drug diffusion and therefore reducing the probability to form bigger nuclei and consequently large NCs. In consequence, for all experiments, MeOH/H_2_O volume ratio of 1.5/10 was used to produce the ETO NC nanosuspensions repeatably, after the evaporation/rehydration process in 6 mL water with 10 mg of F-127 (P2) (0.2% w/v).

**Fig. S1.** ETO NCs mean diameter size measured by dynamic light scattering versus MeOH/H_2_O volume ratio after redispersion in water.

Influence of the F-127 (P1)/ETO weight ratio

MeOH solution of ETO (total mass of ETO : 2.5 mg) has been injected in water with various amounts of F-127 (P1) comprised between 0 and 20 mg, followed by a complete evaporation and redispersion in 6 mL water with 10 mg of F-127 (P2) (0.2% w/v) to reinforce the NC’s stability in solution. In that case, the ETO NC size has been evaluated to explore the impact of the weight ratio F-127 (P1)/ETO on the nanocrystal size (Fig. S2).

**Fig. S2.** ETO NCs mean diameter size measured by dynamic light scattering versus F-127 (P1)/ETO weight ratio after redispersion in water.

The amount of stabilizing agent added before evaporation influences the size of ETO NCs in suspension. In particular, when 5 mg or less of F-127 for 2.5 mg total ETO is used, NC size of approximately 100 nm are obtained, contrary to the suspensions prepared with more than 5 mg of F-127 (P1), where NCs have a size ranging from 450 nm to 600 nm. The results obtained from these precipitation experiments showed that the stabilizing agent F-127 (P1), is not needed in the aqueous solution for the evaporation step to have small NCs of ETO. These outcomes are counterintuitive as literature concerning NC synthesis mainly used high stabilizer concentration to control drug nucleation and obtain small nanoparticles. Pouretedal worked on the preparation of azithromycin NCs using antisolvent precipitation and studied the size effect of several stabilizers (HPMC, PVP, PVA, PEG) at a concentration range of 0.5 – 5.0% w/v. The obtained results revealed a drastic size diminution with the augmentation of the stabilizer concentration independently of the stabilizer. With Tween 80 at 1 mg/mL, the size distribution was 4,064 nm while at 4.5 mg/mL the size reached 504 nm^2^. Similar conclusions were made by Mansouri *et al.* who prepared ibuprofen NCs with 5 different stabilizers (Triethanolamine, SDS, PVP, SLS, Tween 80); 3,000 nm NCs were synthesized with SDS at 0.1 mg/mL whereas with 1 mg/mL SDS, ibuprofen nanoparticles had a size of around 900 nm^3^. In our study, it has been demonstrated that the NC size reduction could be done without the use of stabilizer during the solvent/antisolvent precipitation due to optimized solvent/antisolvent volume ratio and solvent characteristics. The use of stabilizer was only necessary when redispersing the NCs in water after evaporation (stabilizing agent F-127 (P2)) to prevent NC agglomeration in solution. Besides, adding the stabilizer only in the final step significantly impacted the final drug loading as no stabilizer is present during the evaporation step which usually creates foam that may lead to ETO loss.

Influence of the stabilizer and the water volume redispersion

The NC stabilization/protection was done using poloxamer F-127 which is a non-ionic triblock copolymer comprising a middle hydrophobic block of polypropylene glycol (PPG) bordered by two hydrophilic blocks of PEG. Numerous parameters influence the NC stability, the polymer molecular mass, the hydrophobic/hydrophilic ratio of the chain, the nature of the chain functional groups and the polymer conformation^4^. Sharma *et al.* examined the size of indomethacin NCs produced by wet milling and high pressure homogenizer (HPH) using two dissimilar surfactants, Polyvinylpyrrolidone-K25 (PVP) and F-127; the results indicated a surface modification and a size difference between NCs according to the nature of the polymer^5^. Lee *et al.* also evidenced the impact of the hydrophobic/hydrophilic polymer ratio on the NC steadiness by producing a polymer with the same hydrophobic part but diverse hydrophilic groups. The study concluded that the polymer hydrophobicity is a striking parameter to the particle stabilization. Indeed, the polymer should contain a minimum of 15% hydrophobic moieties to present efficient stabilization, as the adsorption is better on the NC surface in such a case^6^. Hence, Pluronic F-68 and F-127 can be considered as stabilizing agents for ETO NCs since their hydrophobic moieties represent ~16% and ~25%, respectively^7,8^. The NC physical state, size, shape and morphology should be chosen in accordance to the application, target and route of administration of the drug NCs (oral, ocular, parenteral administration). A fast dissolution of the drug and high saturation solubility are not always needed. Therefore, to go further with the optimization of the NC nanosuspension formulation, the impact of the final concentration of the stabilizer (P2) as well as its nature (F-68 *vs.* F-127) have been studied regarding the NC size over time (Table S1).

***Table S1.*** Diameter mean size in intensity and PDI (value ± SD) of ETO NCs in suspension measured by dynamic light scattering. Comparison of F-127 or F-68 (P2) at 0.03% and 0.08% w/v after redispersion in 6 mL water,
n = 3

| **Formulation** | **Size (nm)** | | **PDI** | **Size (nm)** | **PDI** | **Size (nm)** | **PDI** |
| --- | --- | --- | --- | --- | --- | --- | --- |
|  | **t_0h_** | | | **t_5h_** | | **t_24h_** | |
| ETO NCs F-127 0.03% w/v | | 117 ± 28 | 0.350 ± 0.037 | *274* ± 10 | 0.318 ± 0.010 | *324* ± 7 | *0.358* ± 0.058 |
| ETO NCs F-127 0.08% w/v | 111 ± 36 | | 0.530 ± 0.013 | *282* ± 65 | 0.566 ± 0.049 | *300* ± 115 | *0.697* ± 0.113 |
| ETO NCs F-68 0.03% w/v | 217 ± 3 | | 0.579 ± 0.017 | *268* ± 23 | 0.910 ± 0.127 | Precipitation | Precipitation |
| ETO NCs F-68 0.08% w/v | 175 ± 27 | | 0.357 ± 0.023 | *402* ± 12 | 0.673 ± 0.074 | Precipitation | Precipitation |

It has been found that ETO NCs were better stabilized with 0.03% w/v of F-127 (P2) stabilizing agent as compared to 0.08% w/v for both F-127 and F-68. Moreover, the stability of NCs was found better with F-127 as its affinity (*via* *e.g.* hydrophobic/hydrophilic or van der Waals interactions) allows a better adsorption to the NC surface compared to the F-68. As can be seen on Table S1, the nanosuspension prepared with 0.03% w/v F-127 presents a size of approximately 250 nm after 5 h, and 300 nm at 24 h. The nanosuspension prepared with F-68 has a mean size of 268 nm after 5 h, however the nanosuspension precipitate after 24 h revealed that NCs are not fully stabilized with F-68. The results obtained led to a preferential use of
F-127 during rehydration (P2). The stabilizer concentration used in our study was optimized to be minimal in order to produce a safer formulation and prevent adverse effects. Commonly, in the literature, the stabilizer concentration is above 1% w/v, with a combination of stabilizer such as HPMC/PVP, F-68/PVP or PVP/β-cyclodextrin^9,10^.

**Characterization of etoposide precipitates by transmission electron microscopy (TEM)**

In order to evaluate if CT23 or 3LL cell apoptosis was due to dehydration of the cells caused by precipitation of the solubilized part of ETO on the TEM grid rather than ETO NCs incubation, solutions of Etoposide were analyzed by TEM after dehydration. In that case, we observed needles of etoposide due to ETO precipitation when it is not in its nanocrystalline from. Large objects are obtained since no control of the crystal growth can be achieved in absence of stabilizer.


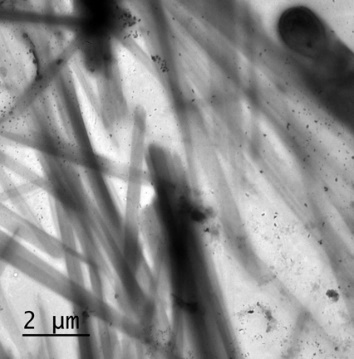

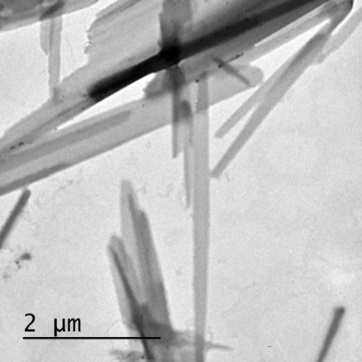


**Fig. S3.** TEM images of ETO crystals shape after dehydration of the Etoposide aqueous solutions.

**Plasma pharmacokinetics of etoposide**

The protein binding *in vivo* on ETO nanocrystals has been apprehended by evaluating ETO/F-127 NCs coated with bovine serum albumin (BSA or Alb.) by comparing the pharmacokinetics of ETO/F-127 and ETO/F-127+Alb. formulations to Toposar and Free ETO formulations. Right after the injection, the Y_0_ is 58.2 and 48.2 µg/mL for ETO/F-127 and ETO/F-127/+Alb. formulations, respectively while 37.2 and 37.6 µg/mL for Toposar and free ETO, respectively (Fig. S4 and Table S3). These differences clearly indicate that the etoposide circulates a certain time as nanocrystals in the bloodstream after the injection of the nanocrystalline formulations, confirming the deviation in the mouse survival data between Toposar and ETO NCs.

**Fig. S4.** Plasma pharmacokinetic after i.v. injection for Free Etoposide, ETO NCs F-127 0.2% w/v, ETO NCs F-127 0.2% w + Alb 0.48% and Toposar. Data are presented as mean ± SD (n=6).

**Table S2.** The y-axis intercept of the one-phase exponential decay at x = 0 (Y_0_), half-life (t_1/2_) and AUC_0-120min_ comparison for Free Etoposide, ETO NCs F-127 0.2% w/v, ETO NCs F-127 0.2% w + Alb 0.48% and Toposar^®^ in plasma. Data are presented as mean ± SD (n=6)

|  | Y_0_  (µg/mL) | Half-life  (min.) | AUC_0-120min_ |
| --- | --- | --- | --- |
| Free Etoposide | 37.6 ± 0.9 | 4.1 ± 0.8 | 378.7 ± 29.2 |
| Toposar | 37.2 ± 1.1 | 5.5 ± 1.1 | 436.6 ± 42.6 |
| ETO NCs/ F-127 0.2% | 58.2 ± 1.9 | 5.5 ± 1.3 | 608.9 ± 66.8 |
| ETO NCs/ F-127 0.2% + Alb 0.48% | 48.5 ± 1.0 | 5.7 ±0.8 | 550.5 ± 37.3 |

**In vitro CT26 and 3LL cytotoxicity**

The cytotoxicity of ETO NCs was investigated in order to define if the efficacy of the drug was not affected by the process of formulation. In Fig S3, no statistical difference could be noted at 48 or 72 h after the incubation of the ETO NCs as compared to the Toposar, independently of the cell line tested.

CT26

3LL

**Fig. S5.** CT26 and 3LL cell viability after 48 h (left) and 72 h (right) for ETO NCs F-127 0.08% w/v and Toposar. Each point represents the mean ± SD (n=3). The data were fit to a sigmoidal dose response model. The inhibitory concentrations for 50% of cells (IC50) were determined as function of control well (doted lines).

**Tissues pharmacokinetics of etoposide**

Tissues biodistribution analysis was performed on mice sacrificed at 45, 60 or 120 min. The main recovery of ETO was found in the lungs and liver for both formulations (Table S2). ETO was not detected in the kidneys or spleen at any time whatever the ETO formulation, thus data for these organs are not shown. For both formulations, the AUC_0-120min_ in the liver was not significantly different (p > 0.05), therefore the biodistribution can be considered equivalent after 120 min. The ETO accumulation in the liver can be explained by the physical state (*e.g.* size) of the nanoparticles but additionally by its recognition by the cells of the mononuclear phagocyte system (MPS) that uptakes and removes exogenous agents^11^.

***Table S3.*** Drug distribution in liver and lungs for ETO NCs F-127 0.08% w/v and Toposar after an i.v. injection at 10 mg/kg in mice. Data were collected at 45, 60 and 120 min. after the injection and are presented as followed in µg of ETO per g of organ ± SD (n=6)

|  | **Etoposide distribution (µg/g)** | | | | | |
| --- | --- | --- | --- | --- | --- | --- |
| **Formulations** | **Liver** | | | **Lungs** | | |
|  | **45 min** | **60 min** | **120 min** | **45 min** | **60 min** | **120 min** |
| ETO NCs/F-127 0.2% w/v | 10.5 ± 9.2 | 4.3 ± 4.9 | – | 51.3 ± 19.9 | 21.8 ± 11.3 | – |
| Toposar | 5.9 ± 4.7 | 6.4 ± 2.3 | – | 86.9 ± 6.9 | 8.9 ± 5.4 | – |

Regarding the lungs, Toposar was not notably greater (p < 0.05), having an AUC_0-120min_ of 2943 µg.min/g, in comparison with ETO NCs /F-127 0.2% w/v that had an AUC_0-120min_ of
2557 µg.min/g (Table S3). For i.v. delivery, rod-shaped nanoparticles between 20 and 150 nm, like ETO NCs, were proved to end up in organs such as the lungs and the liver^12^. Hence, the drug accumulation in the lungs could be elucidated by the size of NCs comprised between 100 - 300 nm. Undeniably, particle size and shape change the nanoparticle biodistribution, the circulation lifespan related to the recognition by the MPS, and the particle extravasation in the blood stream^13^. In conclusion, it is interesting to notice that ETO NC formulations have similar elimination properties like Toposar.

**Table S4.** AUC_0-120min_ comparison for ETO NCs F-127 0.2% w/v and Toposar in liver and lungs. Data are presented as followed AUC_0-120min_ (µg.min/g) ± SD (n=6)

|  | **Etoposide AUC_0-120min_ (µg.min/g)** | |
| --- | --- | --- |
| **Tissues** | **Toposar** | **ETO NCs/F-127 0.2% w/v** |
| Liver | 416 ± 131 | 517 ± 283 |
| Lungs | 2,943 ± 230 | 2,577 ± 671 |

**Methods**

**Nanocrystal preparation.** The ETO NCs were prepared by the method of antisolvent precipitation. Simply, 2.5 mg of ETO was dissolved in 1.5 mL absolute methanol in a glass vial and slowly injected under agitation (1200 rpm) in 10 mL of water. The solubilized drug was precipitated by evaporating the entire solution using a rotavapor under vacuum for 30 min at 10 mbar. The resulting powder was kept under vacuum to remove any traces of solvent. Then, the powder was hydrated with an aqueous solution containing poloxamer as stabilizer or poloxamer with bovine serum albumin solution, followed by 10 min. sonication using a water-bath sonicator to engineer the NCs solution. The optimal NCs preparation was made with 2.5 mg ETO plus 5 mg F-127. Only the volume of redispersion was changed according to the application. BSA was purchased from Sigma-Aldrich (France).

The NC size in the formulation was measured at 25 °C using a ZetaSizer Nano-ZS of Malvern Instrument (Westborough, Massachusetts, United States). Dispersant (water) properties: refractive index = 1.33, viscosity: 0.8872 cP. The F-127-coated NC size was assessed after the nanocrystallization process. Table 1 shows dynamic light scattering (DLS) measurements of nanoparticle mean diameter in intensity of the ETO NCs synthesized in the study. Experiments were done in triplicates.

The morphological evaluation of ETO crystals obtained after dehydration of solubilized ETO aqueous solution has been investigated by transmission electron microscopy. A drop of the ETO solution was put on a copper grid with Formvar films Cu 200 Mesh. Then, negative staining was performed by adding a drop of uranyl acetate solution (1% w/v). The excess fluid was removed with filter paper. The grids were examined under a transmission electron microscope (JEM-100S, JEOL, Tokyo, Japan) at accelerating voltage of 80 kV.

***In vitro* CT26 and 3LL cytotoxicity.** *In vitro* studies were performed to assess the potential of ETO nanocrystals on cancer cells and to lead future *in vivo* studies. Cytotoxicity studies of ETO NCs/ F-127 0.08% w/v and Toposar (2 mg citric acid anhydrous, 650 mg polyethylene glycol 300, 80 mg polysorbate 80 and 33% v/v absolute ethanol) were tested on CT26 colon cancer and 3LL Lewis lung cancer cells. Protocol is described for CT26, equivalent for 3LL. First, CT26 colon carcinoma cells were cultured in Dubelcco’s modified Eagle’s medium (DMEM) containing 10% fetal bovine serum and penicillin/streptomycin (50 mmol) at 37 °C. Cells were plated at the concentration of 200,000 cells/mL in 96 well plates for 24 h. Then, CT26 cells were incubated with ETO NC formulation and Toposar. After, 48 and 72 h, the tested formulations were removed from wells and cell viability was performed using the colorimetric MTT test^14^. Absorbance was determined at 562 nm in a microplate reader (BioKinetics Reader, EL340). The results are displayed as percentage of viable cells.

**Animals.** BALB/c female mice (6 weeks) were supplied by Janvier Laboratory (Le Genest Saint Isle, France). All of the animals were acclimatized at a temperature of 23 ± 2 ◦C and under light/dark conditions for 4 weeks and provided food and water. All the experiments on rodents were performed in accordance with Guidelines for Care and Use of Laboratory Animals of European directive No. 2010-63, and national guidelines, French decree No. 2013-118. The experiments were also validated by Paris Descartes Ethics Committee for Animal Experimentation, CEEA No. 34 at Paris University under the project number APAFiS #20869.

**Plasma and tissues pharmacokinetics of etoposide.** Thirty-six BALB/c female (6 weeks) mice were used for the determination of the ETO NC concentration over time in the plasma and selected tissues (liver, spleen, kidney, lungs). Two formulations were tested: ETO NCs/ F-127 0.2% w/v and Toposar. Each formulation was given intravenously at 10 mg/kg ETO. Then, retro-orbital blood samples were assessed (200 µL) at 1, 15, 30, 45, 60 and 120 min. and added in an Eppendorf tube containing 20 µL of ethylenediaminetetraacetic acid (EDTA). Tissue samples were taken after mice were sacrificed at 45, 60 and 120 min. in order to have enough drug accumulation in the selected organs and frozen at -80 °C for further HPLC analysis. ETO contained in organs were recuperated by grinding organs in chloroform using Precellys tubes. All organs were washed in water before crushing. Liver and kidneys were crushed in 5 mL of chloroform in 7 mL capacity Precellys tubes while lungs and spleen in 1.2 mL of chloroform in 2 mL capacity Precellys tubes. Samples were centrifuged and chloroform was totally evaporated in glass vials. Dry residues were redispersed in 130 µL of acetic acid 1% in water / methanol mixture as a mobile phase (58/42 v/v) and ready for analysis. The HPLC was set as reversed phase (RP-HPLC, 1260 Infinity, Agilent) with isocratic conditions. The analytical column was standard with a reversed phase C18 (150 mm × 4.6 mm, 2.7 μm, Macherey Nagel). The injected volume was 50 μL for all samples. For the plasma pharmacokinetics experiments, free ETO and ETO NCs/F-127 + BSA have also been tested. Free ETO solution was prepared by solubilizing the same amount of etoposide as in the ETO/NCs/F-127 formulation in a 26.2% w/v of ethanol in aqueous solution (*i.e.* same volume fraction as ethanol in Toposar). ETO NCs/F-127 + BSA was prepared by rehydrating after the antisolvent precipitation the same amount of etoposide as in the ETO/NCs/F-127 formulation with a 0.2% and 0.48% w/v aqueous solution of F-127 and BSA, respectively.

**References**

1. Erdemir, D., Lee, A. Y. & Myerson, A. S. Nucleation of crystals from solution : classical and two-step model. *Acc. Chem. Res.* **42**, 621–629 (2009).

2. Pouretedal, H. R. Preparation and characterization of azithromycin nanodrug using solvent / antisolvent method. *Int. Nano. Lett.* **4**, 103–111 (2014).

3. Mansouri, M., Pouretedal, H. R. & Vosoughi, V. Preparation and characterization of ibuprofen nanoparticles by using solvent / antisolvent precipitation. *Open. Conf. Proc. J.* **1050**, 88–94 (2011).

4. Demetzos, C. in *Pharmaceutical Nanotechnology Fundamentals and Practical Applications, 1^st^ edition.* Adis ed., Athens, Greece (2016).

5. Sharma, P., Denny, W. A. & Garg, S. Effect of wet milling process on the solid state of indomethacin and simvastatin. *Int. J. Pharm.* **380**, 40–48 (2009).

6. Lee, J., Lee, S., Choi, J., Youn, J. & Ahn, C. Amphiphilic amino acid copolymers as stabilizers for the preparation of nanocrystal dispersion. *Eur. J. Pharm. Sci.* **24**, 441–449 (2005).

7. Shubhra, Q. T. H., Oth, J. T. & Gyenis, J. A. Poloxamers for surface modification of hydrophobic drug carriers and their effects. *Polym. Rev.* **54**, 112–138 (2014).

8. Kabanov, A. V., Batrakova, E. V. & Alakhov, V. Y. Pluronic® block copolymers as novel polymer therapeutics for drug and gene delivery. *J. Control. Release* **82**, 189–212 (2002).

9. Khan, S., Matas, M. De, Zhang, J. & Anwar, J. Nanocrystal preparation: low-energy precipitation method revisited. *Cryst. Growth Des.* **13**, 2766–2777 (2013).

10. Yarraguntla, S. R., Enturi, V., Vyadana, R. & Bommala, S. Formulation and evaluation of lornoxicam nanocrystals with different stabilizers at different concentrations. *Asian J. Pharm.* **10**, 198–207 (2016).

11. Couvreur, P. & Malvy, C. eds., *Pharmaceutical Aspects of Oligonucleotides*, Taylor & Francis Ltd (1999).

12. Blanco, E., Shen, H. & Ferrari, M. Principles of nanoparticle design for overcoming biological barriers to drug delivery. *Nat. Biotechnol.* **33**, 941–951 (2015).

13. Lavoie, P. M. & Levy, O. in *Mononuclear Phagocyte System, 5^th^ Edition*, Elsevier Inc., (2016).

14. Breton, M., Leblond, J., Johanne, S., Midoux, P., Scherman, D., Herscovici, J., Pichon, C. & Mignet, N. Comparative gene transfer between cationic and thiourea lipoplexes. *J. Gene Med.* **12**, 45–54 (2010).
